# Sutures ultrasound: useful diagnostic screening for posterior plagiocephaly

**DOI:** 10.1007/s00381-021-05324-3

**Published:** 2021-08-28

**Authors:** Silvia Marino, Martino Ruggieri, Lidia Marino, Raffaele Falsaperla

**Affiliations:** 1 Unit of Pediatrics and Pediatric Emergency, AOU “Policlinico”, PO “San Marco”, University of Catania, Catania, Italy; 2 Unit of Rare Diseases of the Nervous System in Childhood, Department of Clinical and Experimental Medicine, Section of Pediatrics and Child Neuropsychiatry, University of Catania, Catania, Italy; 3 Neonatal Intensive Care Unit [NICU], AOU “Policlinico”, PO “San Marco”, University of Catania, Catania, Italy

**Keywords:** Plagiocephaly, Skull deformities, Craniosynostosis, Craniostenosis, Suture ultrasound

## Abstract

**Purpose:**

Posterior plagiocephaly (PP) is a common clinical condition in pediatric age. There are two main causes of PP: postural plagiocephaly and craniosynostosis. Early diagnosis is important, as it prevents neurological complications and emergencies. Diagnosis in the past was often made late and with imaging tests that subjected the infant to a high radiation load. Suture ultrasound does not use ionizing radiation; it is easy to perform, allows an early diagnosis, and directs toward the execution of the cranial 3D-CT scan, neurosurgical consultation, and possible intervention. The aim of the study is to describe the high sensitivity and specificity of suture ultrasound for the differential diagnosis between plagiocephaly and craniosynostosis.

**Methods:**

We reported our prospective experience and compared it with the data in the literature through a systematic review. The systematic review was conducted on electronic medical databases (PubMed, Embase, Cochrane Library, Scopus, and Web of Science) evaluating the published literature up to November 2020. According to Preferred Reporting Items for Systematic Reviews and Meta-ANALYSES (PRISMA statement), we identified 2 eligible studies. Additionally, according to AMSTAR 2, all included reviews have been critically rated as high quality. A total of 120 infants with abnormal skull shape were examined in NICU. All underwent clinical and ultrasound examination.

**Results:**

Of the total, 105 (87.5%) had plagiocephaly and 15 dolichocephaly/scaphocephaly (12.5%). None of these had associated other types of malformations and/or neurological disorders. The synostotic suture was identified ultrasonographically in 1 infant and subsequently confirmed by 3D CT scan (100%).

**Conclusion:**

Cranial sutures ultrasonography can be considered in infants a selective, excellent screening method for the evaluation of skull shape deformities as first technique before the 3D CT scan exam and subsequent neurosurgical evaluation. Cranial suture ultrasonography should be considered part of clinical practice especially for pediatricians.

**Supplementary information:**

The online version contains supplementary material available at 10.1007/s00381-021-05324-3.

## Introduction

The term plagiocephaly derives from the Greek *plagio* (oblique, twisted, or slant) and *kephale* (head), it is defined as an asymmetric shape of the skull with a unilateral or more flattening. Plagiocephaly has been distinguished in two groups: synostotic plagiocephaly (SP) and positional plagiocephaly (PP) [1]. Sutures are constituted by tough, elastic fibrous tissue which delimits the bones from one another. The sutures normally remain flexible and do not fuse together until around age of 2 years. SP is a form of non-syndromic or syndromic craniosynostosis due to premature fusion of one or more cranial suture. Positional molding is a distinct entity caused by intrauterine restriction or by postnatal deformational forces [2–9]. Following the “Back to sleep” campaign introduced by American Heart Association to prevent SIDS, the incidence of positional plagiocephaly has increased significantly [10]. Upon discharge from the neonatal intensive care unit, it is a condition that is always evident especially for infants who have required long periods of hospitalization. This condition not only raises concern in parents with additional specialist outpatient visits but can also hide synostotic plagiocephaly (left or right lambdoid or both). Suture ultrasound represents a non-invasive and safe technique that allows a filter for those infants who subsequently have to undergo three-dimensional computed tomography (3D-CT) and then neurosurgery consultation. It therefore avoids submitting the newborn to unnecessary radiation. Ultrasound (US) has been proposed to assist practitioners in diagnosing skull deformities with excellent efficiency [11–25]. However, few studies have been conducted to date, and this technology is still now not proposed as a common diagnostic practice. The purpose of this study is to report the results on infants presenting with posterior plagiocephalies with the use of the cranial suture ultrasound. Data collected by systematic literature review is also reported.

## Material and methods

We conducted a single observational study of infants aged 0–28 days, admitted from March 2020 to March 2021 to the Neonatal Intensive Care Unit (NICU). The examination, performed by the same operator, was carried out by means of Esaote MyLabAlpha ultrasound system with linear probe 3–22 MHz. The probe was positioned perpendicular along the entire course of the cranial suture. The main cranial sutures analyzed were the following: metopic, right and left coronal, sagittal, and right and left lambdoid. The suture was considered normal or patent when the hypoechoic bone gap was identified; otherwise, synostotic [12, 13]. In case of synostotic suture, the infants underwent a 3D-CT scan and neurosurgical consultation.

### Search strategy

We searched on medical electronic databases (PubMed, Embase, Cochrane Library, Scopus, and Web of Science) evaluating the published literature up to November 2020. According to Preferred Reporting Items for Systematic Reviews and Meta-Analyses (PRISMA statement), the MeSH terms “ultrasound” AND “craniostenosis” AND/OR “posterior plagiocephaly” were used. We included all prospective or retrospective observational studies, case series, reviews, and case reports. Of these studies, we selected articles with “Infant: birth–23 months,” and only articles in “English” were selected "(Flow Diagram PRISMA)". Two authors independently evaluated articles for inclusion, extracted data, and assessed quality of evidence using AMSTAR 2 (A Measurement Tool to Assess Reviews). According to AMSTAR 2, all included reviews were rated critically as being of high quality.

## Results

### Literature systematic review

The search resulted in a total of 146 studies, of which 97 were eligible studies for “Infant (birth–23 months)” and English language. Considering the last 10 years, from year 2011, we found 43 eligible articles for our research. We reviewed pediatric scientific articles on suture ultrasound in posterior plagiocephaly or craniostenosis diagnosis in infant and, at the same time, high-resolution sonography technique and comparison with 3D-CT scan. We excluded 41 full-text articles because all 41 do not describe the relationship between the ultrasound and posterior plagiocephaly features. After considering the selection criteria, ultrasound in posterior plagiocephaly, and/or craniostenosis, we included in the study 2 articles. Those studies were one retrospective and other a prospective study (Table 1).

**Table 1:** Review literature with our experience: sensitivity and specificity suture US

**Literature**	*Sze et al. (2003)*	*Regelsberger et al. (2006)*	*Simanovsky *et al*. (2009)*	*Krimmel et al. (2012)*	*Alizadeh et al. (2013)*	*Linz et al. (2015)*	*Rozovsky et al. (2016)*	*Pogliani et al. (2017)*	*Hall et al. (2017)*	*Proisy et al. (2017)*	*Our study*
**Sample**	41	26	24	54	44	269	126	196	60	40	120
**Mean age (months)**	7.7	(2–7)	4.3	6.0	5.7	6.41	(0–12)	4	4.6	5.2	0.50
**Synostosis**	2	26	8	7	31	8	8	28	7	30	1
**Sensitivity US %**	100	100	100	71.4	96.9	100	100	100	100	100	100
**Specificity US %**	89	100	100	95.7	100	100	100	86	86	100	100

### Our experience

Data from 120 infants were collected, and 82 (68.3%) of them were male. Median clinical examination age was 15.1 days. Moreover, they presented a gestational age from 30 to 36 weeks.

#### Clinical features

One hundred five (87.5%) presented with PP and 15 (12.5%) had dolichocephaly/scaphocephaly. Clinically posterior plagiocephaly presented occipital flattening with a parallelogram-like head shape and dolichocephaly and overlapping parietal bones, except for one case. None of these had associated other types of malformations and/or neurological disorders.

#### Suture US

In 119, ultrasound showed the patency of the main cranial sutures, diagnosing a postural anomaly. In the case of dolichocephaly, an early fusion of sagittal suture was noted and underwent 3D-CT scan, and the closure of the suture was confirmed (Flow Diagram PRISMA).

#### Follow-up

Follow-up was performed by clinical exam, ophthalmological evaluation, and transfontanellar and suture ultrasound after 1, 3, 6, and 12 months from discharge. It was observed a gradual resolution of cranial abnormality.

## Discussion

Plagiocephaly refers to an asymmetrical, deformity with unilateral skull flattening. Classically, the anomaly is distinct in synostotic and not, the latter noted also as positional plagiocephaly (PP). PP is linked to intrinsic factors and intrauterine constraints as happens with multiple births, large size for gestational age, oligohydramnios, breech and transverse position, and congenital torticollis [26]. Preterm births are particularly at risk of PP, and there is an inverse correlation between preterm age and PP risk [2]. Causes are the soft skull and placement within the thermal cradle. PP is a self-limiting clinical condition, where timely cranial osteopathy favors the resolution of the anomaly with early treatment [25, 26]. In these cases, the head assumes the typical flattened shape posteriorly, or brachycephaly. On the contrary, in SP, the absence of the physiological patency of cranial sutures represents an obstacle to the normal development of the encephalic structures and the facial muscles, leading to esthetic, ocular, and chewing problems, but above all, intracranial hypertension. Therefore, early and prompt diagnosis is crucial to prevent possible damage. Linz et al. in a prospective study examined 411 children with non-syndromic skull abnormalities and defined clinical parameters that could differentiate between positional and lambdoid synostosis (LS). Eight cases of unilateral LS and 258 cases of positional plagiocephaly were confirmed by suture ultrasound. Linz et al. suggest that ipsilateral occipital flattening, a downward shift of the ipsilateral ear, and a parallelogram-like head shape in the posterior view can be used as diagnostic tools for lambdoid synostosis [27]. Di Rocco in a retrospective analysis enrolled 165 patients, ranging from 0 to 18 years of age, submitted to a skull CT scan for head trauma. The 25% present a positional posterior plagiocephaly. The results were analyzed with different age group, with a high prevalence in adolescents. They hypothesize, in contrast with the literature, that positional posterior plagiocephaly does not correct spontaneously in all children, with a prevalence of deformational plagiocephaly being more common than usually reported and that may persist at a late age [28]. It is therefore important, at an early stage, cranial screening by clinical examination and, above all, with suture ultrasound. Over the years, there has been an evolution in diagnostic imaging. From the cranial radiography, burdened by radiation and poor sensitivity and specificity, we passed directly to the gold standard represented by cranial CT with 3D reconstruction, which, as known, is not free from adverse effects such as sedation and exposure to ionizing radiations. Recently, ultrasound is making its way into several clinical fields. In 1997 and 1998, Soboleski et al. were the first to demonstrate that high-resolution ultrasound could show normal cranial sutures, and a year later, they described the sonographic appearance of synostotic cranial sutures and diastasis in case of intracranial hypertension [12, 13]. The cranial suture US represents a valid and appropriate technique for the evaluation of the cranial sutures and indication, in doubtful cases, to make use of a 3D CT scan and neurosurgical specialist advice. However, to date, there are few studies in the literature that correlate cranial suture US with other imaging investigations, but it is sufficient to use the ultrasound technique in clinical practice. Results on cranial suture US taken by the literature are summarized in Table 1. Proisy et al. reported, a wide study on craniostenosis showing how ultrasound in routine practice can contribute to the diagnosis of craniosynostosis analyzing the results obtained by reviewing the literature [14, 15]. In 2003, Sze et al. conducted a prospective study on 41 children who had already undergone cranial CT and subsequently cranial suture ultrasound for the study of blinded posterior plagiocephaly by 3 radiologists. In this case, the ultrasound showed a sensitivity of 100% and a specificity of 86–92%. The false positive results were due to images captured with incorrect positioning of the probe on the cranial vault and not on the suture [16]. Simanovsky et al. in 2009 evaluated 24 newborns with an average age of 4.3 months by ultrasound; in 23, they had a correct diagnosis while in only one case, this was inconclusive to refer to partial synostosis. Also in this case, a sensitivity and specificity of 100% was observed [18]. Krimmel et al. in 2012 instead reported a sensitivity and specificity of 71% and 95% respectively. By studying 54 children, 7 real synostoses were found while in 2 children, the ultrasound was inconclusive as the CT scan demonstrated a partial fusion [19]. Alizadeth et al. conducted a study of cranial sutures in 43 infants, and the diagnosis was consistent with CT. Only in a 7-month patient did the diagnosis escape in a newborn with a positive family history, in which CT scan detected primary compound craniosynostosis, involving both sagittal and metopic sutures presenting scaphocephaly. In the CT scan, a 4-cm-long bridge of the suture was noted. The sensitivity and specificity of ultrasound compared to CT scan were 96.9% and 100%, respectively. There was no significant difference in the diagnostic accuracy of ultrasound between girls and boys and between infants younger than 6 months and children older than 6 months [20]. Pogliani et al. in 2013 showed the high sensitivity and specificity of cranial sutures with ultrasound and obtained the 100% and 86% of the results, respectively. They studied a cohort of 196 children; in 30, the synostosis was diagnosed by ultrasound, except for 2,and in the remaining 29, the diagnosis was confirmed by CT. Twelve patients with severe cranial deformity and negative ultrasound underwent CT, which confirmed the ultrasound results in all of them, except for one case in a child affected by bitemporal synostosis. Pogliani et al. also observed an inverse correlation between the adequacy of ultrasound and age; the younger the patient, the more specific and adequate the ultrasound result is. The year of age can represent a limit for which the ultrasound may be inconclusive [22, 23].

**Fig. 1:**
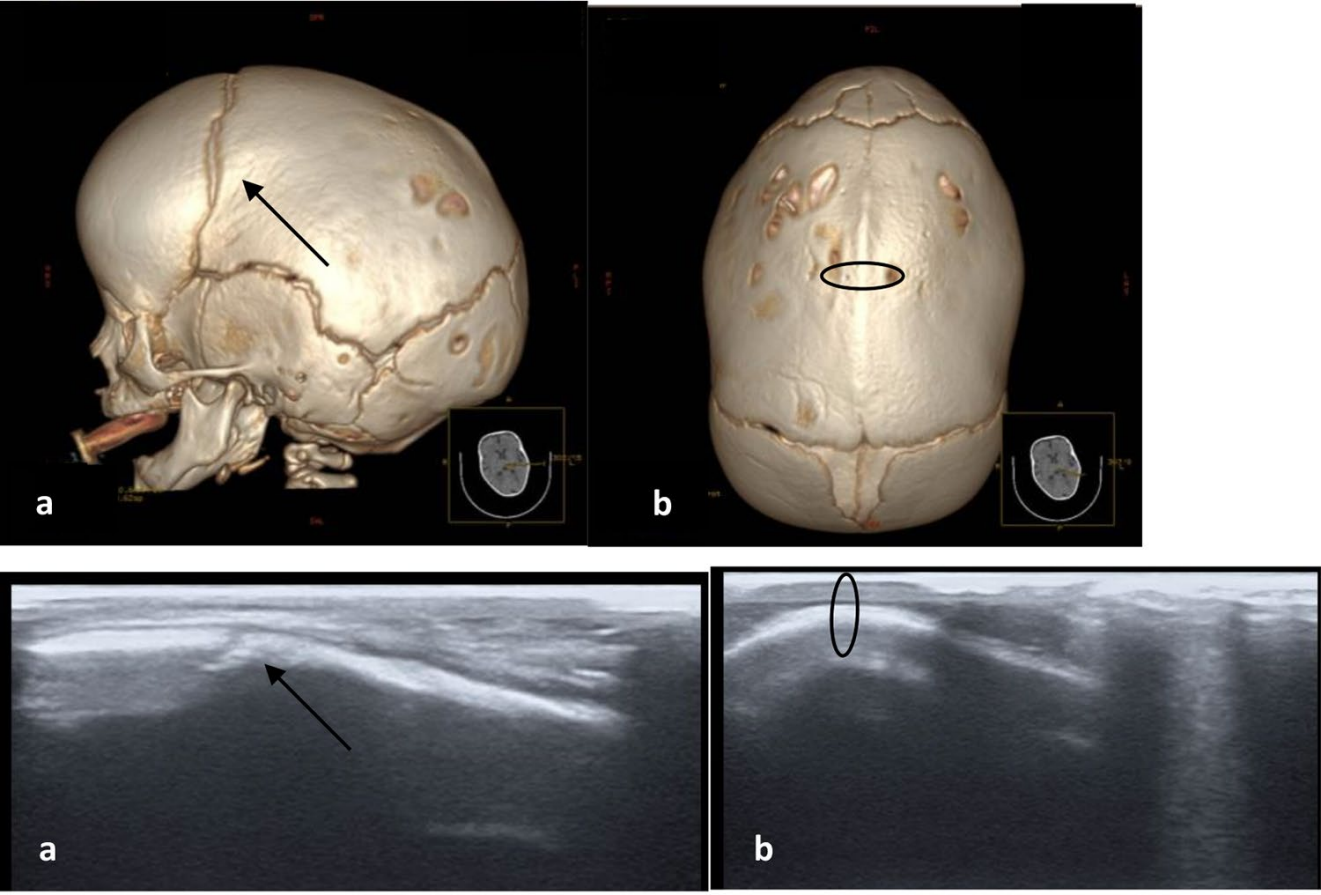
Scaphocephaly. **a** 3D-CT lateral view: skull shape with elongated cranium, with patent coronal suture (arrow); **b** 3D-CT superior view: showing total sagittal synostosis (ring), with corresponding US images

In the our study involving 120 infants with skull deformities, patency of all sutures with the typical anechoic gap was observed, by ultrasound, in all except one child, confirmed by 3D-CT. This is in keeping with the literature. According with studies, the finding of synostotic lambdoidea is rare, and the data suggests that incidence ranges from 1 to 3% [29]. The unique anomaly was then confirmed on 3D-CT. The infant underwent subsequent neurosurgical consultation and were submitted to surgery correction. The ultrasound of the cranial sutures also in this study showed a high specificity and sensitivity, equal to both 100% (Fig. 1). A quarterly clinical and ultrasound follow-up was conducted in infant clinical exam, ophthalmological evaluation, and transfontanellar and suture ultrasound after 1, 3, 6, and 12 months from discharge, with normalization of the cranial shape. Some concern of the use of cranial suture ultrasound may be due to the age; an older child may cause difficulty in performing the ultrasound examination due to poor tolerance, as well as thick hair that will increase a reduction in the transmission of acoustic waves and an increase in the execution time employed. In favor of ultrasound, besides the absence of sedation and ionizing radiation, are the ease of execution and repeatability. These are superficial and identifiable structures by means of the two landmarks represented by the anterior and posterior fontanelles. Finally, it allows to not underestimate the possibility of immediate feedback and an interview with the parents present.

## Conclusion

Suture ultrasound is a convenient, simple, non-invasive, low-cost, and rapid imaging technique. From our study and from the systematic review of the literature, ultrasound can be considered a reliable method as a screening to differentiate plagiocephaly from craniosynostosis. Our experience supports and extends the data present in the literature. Ultrasound should be used in all cases of plagiocephaly with or without other associated symptoms and/or malformations, and it should be introduced into clinical practice.

## Supplementary information

Below is the link to the electronic supplementary material.

Supplementary file1 (DOC 61 KB)

Supplementary file2 (DOCX 36 KB)
