# Supplementary material for: Sutures ultrasound: useful diagnostic screening for posterior plagiocephaly
Source: Childs Nerv Syst. 2021 Aug 28;37(12):3715–20. doi: 10.1007/s00381-021-05324-3 (PMC8604816; doi:10.1007/s00381-021-05324-3)
Supplement: Supplementary file 1 — Supplementary file1 (DOC 61 KB) [file 381_2021_5324_MOESM1_ESM.doc]

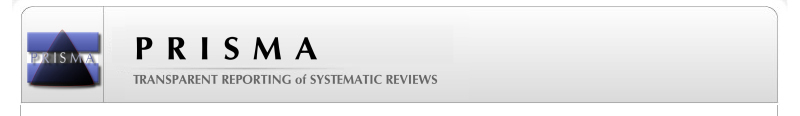
**PRISMA 2009 Flow Diagram**

**Screening**

**Included**

**Eligibility**

**Identification**

Records identified through electronic database searching (PubMed, Embase, Cochrane Library, Scopus, and Web of Science)

(n =146)

Filters:

- “Infant: birth-23 months “
- “English”

Records screened
(n = 97)

Records excluded
(n = 54) because were published before 2011

Full-text articles assessed for eligibility
(n = 43)

Full-text articles excluded, with reasons
(n = 41)

All article not describe relationship among ultrasound and posterior plagiocephaly and/or craniostenosis:

- Described treatment (n=16)
- Describe ophthalmological features (n=1)
- Syndromic case reports (n=2)
- Described others arguments (n=22)

Studies included in qualitative synthesis
(n = 2)
