# Supplementary material for: Sutures ultrasound: useful diagnostic screening for posterior plagiocephaly
Source: Childs Nerv Syst. 2021 Aug 28;37(12):3715–20. doi: 10.1007/s00381-021-05324-3 (PMC8604816; doi:10.1007/s00381-021-05324-3)
Supplement: Supplementary file 2 — Supplementary file2 (DOCX 36 KB) [file 381_2021_5324_MOESM2_ESM.docx]

**Flow chart 1: Diagnostic approach for infant**

**Positional Plagiocephaly**

**Synostotic Suture**

**3DCT SCAN**

**Neurosurgical consultancy and possible surgical correction**

**Fused/Synostotic**

**Unclear**

**Open/Patent**

**All infant with and without posterior plagiocephaly**
